# Nuclear components and dynamics during plant innate immunity

**DOI:** 10.3389/fpls.2013.00481

**Published:** 2013-11-22

**Authors:** Susana Rivas, Laurent Deslandes

**Affiliations:** ^1^Laboratoire des Interactions Plantes-Microorganismes, Institut National de la Recherche Agronomique, UMR441Castanet-Tolosan, France; ^2^Laboratoire des Interactions Plantes-Microorganismes, Centre National de la Recherche Scientifique, UMR2594Castanet-Tolosan, France

**Keywords:** plant cell nucleus, immune receptor, plant immunity, transcription factors, defence regulator, effector, nucleo-cytoplamic trafficking, hormone signaling

In plants, efficient immune responses against microbial infection depend on the ability to rapidly couple pathogen recognition to downstream signaling responses. In this context, plant immunity requires highly dynamic responses that involve multiple organelles during the recognition and signaling events associated with defense. Nuclear dynamics play a critical role in plant immunity based on the growing number of reports revealing that nuclear localization of pathogen effectors, plant disease resistance proteins, and key plant components, including transcription factors and regulators, are essential for immunity. This Research Topic provides an overview of the current knowledge about the importance of nuclear components—both from the “microbial side” and from the “plant side”—and nuclear dynamics during the establishment of plant immune responses.

Mutations in plant cellular factors involved in the transport of macromolecules through the nuclear envelope compromise plant resistance signaling, underlining the importance of nucleocytoplasmic trafficking during plant innate immunity. The Mini-Review article by Gaouar and Germain (2013) describes the importance of nuclear mRNA export during plant immune responses whereas Wirthmueller et al. (2013) discuss importin-α-mediated nuclear translocation and how microbial effectors may compete with host cargo proteins for nuclear uptake.

Following their delivery into plant cells, a significant number of effector proteins from different pathogenic microorganisms, including viruses, oomycetes, fungi, nematodes, and bacteria, are targeted to the nucleus by co-opting the host nuclear import machinery. This suggests that effectors may manipulate host transcription or directly target essential host nuclear components for the benefit of the pathogen. Indeed, pathogen-induced transcriptional regulation in host cells plays a crucial role in the establishment of plant defense and associated plant cell death responses. Several articles in this Research Topic highlight these ideas. Quentin et al. (2013) describe effectors from plant parasitic nematodes that target host nuclei and possibly interact with nuclear proteins to establish feeding cells in infected plants. In their Original Research article, Ma et al. (2013) show that nuclear localization of the Avr2 effector from the xylem-colonizing fungus *Fusarium oxysporum* is required to trigger I2-mediated resistance in tomato plants, whereas Stam et al. (2013) show the diversity of nuclear functions of CRN effectors from the oomycete *Phytophthora infestans*. Finally, the Opinion article by Noël et al. (2013) discusses recent advances in predicting target sequences of nuclear-targeted TAL effectors from the plant pathogenic bacteria of the genus *Xanthomonas.*

It has been estimated that about 25% of *Arabidopsis* genes are transcriptionally regulated in response to pathogen infection and a significant number of transcription factors are involved in the defense gene regulation. Raffaele and Rivas (2013) review our current knowledge of the transcriptional control of plant defenses with a focus on the MYB family of transcription factors and, within this family, the *Arabidopsis* MYB protein AtMYB30, which is a positive regulator of disease resistance. The Review article by Gimenez-Ibanez and Solano (2013) discusses the nuclear crosstalk of jasmonate and salicylate signaling with other hormone pathways during the fine-tuning of a robust plant defence response.

Spatial restriction of immune receptors and defense regulators by the nuclear envelope as well as their stimulus-induced nuclear translocation provide an important mechanism for defense regulation, as their level of nuclear accumulation determines the magnitude of the defense response. In addition, nuclear translocation of effectors may also affect subcellular localization of their cognate resistance proteins in a process that is essential for plant immunity. Bhattacharjee et al. (2013) review nuclear functions of different immune receptors and associated proteins, including transcription factors and defence regulators. Chang et al. (2013) discuss how nucleo-cytoplasmic partitioning of the barley MLA immune receptor triggers downstream transcriptional responses, thereby providing an efficient connection between pathogen perception and the plant immune response. Finally, in their Original Research Article, Heidrich et al. (2013) provide evidence that the *Arabidopsis* WRKY domain-containing immune receptor RRS1 contributes to temperature-conditioned autoimmune responses conferred by a second nuclear immune receptor, RPS4. These data suggest that RPS4 engages RRS1 to direct defence signaling.

In summary, recent findings from our rapidly evolving field situate the nucleus at the forefront of the mutual recognition between plants and pathogens. Integrating the knowledge on immunity-associated nuclear events within the outlook of whole cellular dynamics represents an exciting perspective for future research.
